# USP16 regulates castration-resistant prostate cancer cell proliferation by deubiquitinating and stablizing c-Myc

**DOI:** 10.1186/s13046-021-01843-8

**Published:** 2021-02-05

**Authors:** Jianchao Ge, Wandong Yu, Junhong Li, Hangbin Ma, Pengyu Wang, Yinghao Zhou, Yang Wang, Jun Zhang, Guowei Shi

**Affiliations:** Department of Urology, The Fifth People’s Hospital of Shanghai, Fudan University, No. 801, Heqing Road,Minhang District, Shanghai, 200240 People’s Republic of China

**Keywords:** USP16, C-Myc, Deubiquitinase, Prostate cancer

## Abstract

**Background:**

c-Myc, a well-established oncogene, plays an important role in the initiation and progression of various cancers, including prostate cancer. However, its mechanism in cancer cell remains largely unknown and whether there exist a deubiquitinase targeting c-Myc also remains elusive.

**Methods:**

Bioinformatic analysis and shRNA screening methods were used to identify potential deubiquitinases that correlate with c-Myc gene signature. Cell proliferation and viability were measured by Cell-Counting-Kit 8 and colony formation assays. A mouse xenograft model of PC3 cells was established to confirm the function of USP16 in vivo. The interaction between USP16 and c-Myc protein was assessed by co-immunoprecipitation and protein co-localization assays. Immunohistochemistry staining was performed to detect the expression of USP16, Ki67, and c-Myc in xenograft tissues and clinical tumour tissues. Furthermore, the correlation between USP16 and c-Myc was confirmed by RNA sequencing.

**Results:**

Functional analyses identified USP16, known as a deubiquitinase, was strongly correlated with the c-Myc gene signature. Depletion of USP16 was shown to significantly suppress the growth of PCa cells both in vitro and in vivo. Co-immunoprecipitation and ubiquitination assays confirmed that USP16 served as a novel deubiquitinase of c-Myc and overexpression of c-Myc significantly rescued the effects of USP16 disruption. Immunohistochemistry staining and RNA-seq tactics were further used to confirm the positive correlation between USP16 and c-Myc expression. Expression of USP16 in human PCa tissues was higher than that seen in normal prostate tissues and its high expression was found associated with poor prognosis.

**Conclusions:**

USP16 serves as a novel deubiquitinase of c-Myc. Downregulation of USP16 markedly suppressed PCa cell growth both in vitro and in vivo. USP16 regulates PCa cell proliferation by deubiquitinating and stabilizing c-Myc, making it a potential therapeutic candidate for the treatment of PCa.

**Supplementary Information:**

The online version contains supplementary material available at 10.1186/s13046-021-01843-8.

## Background

Prostate cancer (PCa) is one of the leading causes of death in men worldwide [1]. Due to the significance of androgen receptor (AR) signalling in tumourigenesis of PCa [2], most patients with primary PCa receive androgen deprivation treatment (ADT) as their first-line therapy [3]. Although ADT could achieve strong response at the early stage of PCa treatment, almost all the patients relapse into castration-resistant prostate cancer (CRPC), which is considered as the latest stage of PCa and almost incurable, within 18–24 months [4]. As a result, patients with CRPC were forced to endure chemotherapy, such as docetaxel [5], which would cause severe side effects and deteriorate their quality of life. Hence, novel therapeutic strategies are in an urgent need for PCa, especially CRPC treatment.

The protein product of pro-oncogene *MYC* is a potent transcription factor that regulates the transcription of at least 15% of the entire genome [6], governs a diverse array of biological processes including cell proliferation [7], metabolism [8], protein translation [9], and cell-cycle progression [10]. Moreover, *MYC* has also been found as one of the key drivers of CRPC and neuroendocrine prostate cancer (NEPC) pathogenesis [11]. c-Myc is frequently overexpressed in CRPC and its expression is correlated with poor outcomes [12], while N-Myc drives NEPC progression from human prostate epithelium [13]. It has been reported that reduction of *MYC* levels in mice benefits multiple organs and promotes longevity [14].

Ubiquitination is a tightly regulated post-translational modification that could influence a wide range of physiological and pathological processes [15]. Deubiquitinases (DUBs) can reverse ubiquitination process by removing ubiquitin molecule from target proteins [16]. *MYC* is a short-lived protein and its stability is precisely regulated by the ubiquitin-proteasome system [17]. Several DUBs are known to regulate *MYC* stabilization including USP7 [18], USP22 [19], USP28 [20], USP36 [21], and USP37 [22]. Furthermore, P22077, a small molecule inhibitor of USP7, was reported to highly suppress growth of N-Myc-amplified neuroblastoma in a xenograft model [18]. Hence, selectively inhibiting DUBs that stabilize *MYC* could be an attractive strategy for the treatment of *MYC*-driven cancers.

Here, we performed a genome-wide screen to identify DUBs that are positively correlated with the c-Myc gene signature in PCa. As a result, USP16 was selected and revealed as a potential regulator of PCa cell proliferation both in vitro and in vivo. Moreover, USP16 was observed to co-localize and interact with c-Myc. In addition, knockdown of USP16 significantly reduced c-Myc abundance at the post-translational level, while overexpression of wild-type USP16, instead of its catalytic-inactive mutant (C205S) [23], stabilized c-Myc. Together, these data confirmed the role of USP16 as a novel c-Myc deubiquitinase, making it a potential therapeutic candidate for the treatment of primary PCa and CRPC.

## Methods

### Cell culture

PC3 and DU145 cell lines were obtained from the American Type Culture Collection (Manassas, VA). PC3 cells were cultured in RPMI1640 medium and DU145 cells were cultured in Dulbecco’s Modified Eagle’s medium (DMEM) supplemented with 10% foetal bovine serum (Gemini, Woodland Hills, CA) and 1% penicillin/streptomycin (Gibco, Grand Island, NY). All cells were maintained at 37 °C in a 5% CO2 humidified incubator.

### Plasmids and transfection

Short hairpin RNA (shRNA) sequences are listed in Additional file 1: Table S1. These sequences were cloned into the pLKO.1 vector, while Flag-tagged USP16 and c-Myc were cloned into the pLVX vector (Clontech 632,187). Plasmids were transfected into HEK293FT cells using PEI 25 K (23966–1; Polysciences, Warrington, PA, USA) according to the manufacturer’s instructions. Stable transformants of PC3 and DU145 cells were isolated in standard medium supplemented with puromycin (5 μg/mL) (Sigma-Aldrich, St. Louis, MO, USA) for 7 days.

The siRNAs were chemically synthesized by Genepharma (Shanghai, China). The sequences of siRNA (5′-3′) are as follows: siNC (UUCUCCGAACGUGUCACGU), siUSP16 (CCGGAAAUCUUAGAUUUGGCUCCUU). Cells were transfected with Lipofectamine 2000 (Invitrogen, Carlsbad, CA) following the manufacturer’s instructions and the culture medium was replaced about 6 h later. Forty-eight h after transfection, the cells were used for further experiments.Cell proliferation and colony formation assay.

Cell growth was measured by Cell Counting Kit-8 (CK04; Dojindo, Kumamoto, Japan) at indicated time points following the manufacturer’s instructions. The cells were seeded in 96-well plates (1000 cells per well). Ten μL CCK8 reagent was added to 100 μL medium in each well and then incubated at 37 °C for 3 h. The absorbance values were detected using a microplate reader (Tecan, Mechelen, Belgium) at 450 nm (A450).

Cells were cultured in 6-well plates (2000–3000 cells per well) in complete medium for 10–14 days depending on colony size. The cells were then fixed with methanol for 20 min and stained with 0.5% crystal violet for 1 h, and images were captured following a twice wash of PBS.

### Reagents and primary antibodies

MG132(52801ES08) and CHX (40325ES03) were purchased from Yeason (Shanghai, China). The following antibodies were used for western blotting: USP16 (A5861; Abclonal), c-Myc (GTX103436; GeneTex), β-actin (sc-47,778; Santa Cruz Biotechnology). Antibodies used for immunohistochemistry: USP16 (HPA021140; Sigma-Aldrich), Ki67 (sc-15,402; Santa Cruz), c-Myc (#ab32072, Abcam). Antibodies used for immunoprecipitation and immunofluorescence: Flag (#30503ES60, Yeason), USP16 (HPA021140; Sigma-Aldrich), c-Myc (13987S, Cell Signaling Technology).

### Immunoprecipitation and immunoblotting

Extracts for immunoprecipitation were prepared using NP-40 lysis buffer containing phenylmethylsulphonyl fluoride. The extracts were then incubated with protein A/G beads supplemented with the indicated antibodies at 4 °C overnight on a rotator. After incubation, beads were washed for three times and boiled in 1× loading buffer. Protein samples were loaded and separated by SDS-PAGE, and then the proteins were transferred to PVDF membranes and incubated with primary antibodies. Membranes were washed with 1× TBST and incubated with anti-mouse or anti-rabbit secondary antibodies for 1 h. Densitometric analysis was conducted using NIH ImageJ software.

### Real-time PCR analysis

RNA was isolated using TRIzol reagent (Invitrogen, Carlsbad, CA, USA) according to the manufacturer’s instructions. RNA was then reverse transcribed into cDNA using a PrimeScript 1st Strand cDNA Synthesis Kit (6110A; TaKaRa, Kyoto, Japan). qRT-PCR was performed using TB Green Premix ExTaq (Tli RNaseH Plus) (RR420; TaKaRa) on an ABI7500 System (Applied Bio Systems, Foster City, CA, USA). The relative expression levels of genes were calculated using the 2 − ΔΔCt method. GAPDH was used as an internal control for qRT-PCR. The sequence of primers used to amplify the target genes are listed in Additional file 1: Table S1.

### Animal experiments

Approximately 1 × 10^6^ PC3 cells infected by a lentivirus were mixed with Matrigel (volume, 1:1; 356,234; Corning, NY) and subcutaneously implanted into six-week-old male nude mice (*n* = 9). All mice were sacrificed after 8 weeks, at which point the tumours were dissected and weighed. The xenografts were paraffin-embedded for haematoxylin-eosin and immunohistochemical staining. The protocol was approved by the Institutional Animal Care and Use Committee of Shanghai Veterinary Research Institute.

### IHC staining

Tissue sections were deparaffinised in xylene solutions followed by rehydration in graded ethanol. After this, slides were incubated in 3% hydrogen peroxide for 10 min and then boiled in citrate solution (pH = 6) in a microwave for 20 min and cooled to room temperature. The slides were then blocked in an appropriate blocking solution for 30 min and subsequently incubated with primary antibodies overnight at 4 °C. The final IHC scores = intensity score × percentage score. Intensity was scored according to staining intensity (0: negative, 1: weak, 2: moderate, and 3: strong); percentage score was evaluated based on the percentage of stained cells (0: 0%, 1: 1–25%, 2: 26–50%, 3: 51–75%, and 4: 76–100%).

### Immunofluorescence

Cells were seeded at a density of 2 × 10^4^ cells/well in a 24-well plate and cultured for 24 h. After this, the cells were fixed with paraformaldehyde, permeabilized with 0.1% Triton X-100 for 10 min, incubated with the primary antibodies at 4 °C overnight, and then incubated with fluorescent-labelled secondary antibodies for 1 h. The nuclei were counterstained with DAPI. Cell images were captured with a fluorescence microscope (Nikon Eclipse E200).

### RNA sequencing and expression analysis

Total RNA extracted from indicated groups of PC3 cells was subjected to RNA sequencing (RNA-seq) performed by Majorbio Biopharm Technology (Shanghai, China). Expression profiles were obtained using the Majorbio Cloud Platform. The sequence data have been uploaded to Gene Expression Omnibus (GEO, GSE160818).

Gene expression datasets of human PCa samples were downloaded from NCBI-GEO (https://www.ncbi.nlm.nih.gov/geo/). Gene set enrichment analysis (GSEA) was performed using software provided by the Broad Institute (http://www.broadinstitute.org/gsea/index.jsp). The permutation type was “phenotype”, and the genes were ranked based on Pearson correlation score.

### Statistical analysis

All statistical analyses were performed using GraphPad Prism software (version 7; GraphPad Software, La Jolla, CA). Quantitative data obtained from experiments with biological replicates are presented as mean ± SD. Pearson correlation and linear regression analysis were performed. Tumor onset analysis was performed with log-rank tests. Two-tailed Student’s t-test was used to analyze the quantitative data and *p* values < 0.05 were considered statistically significant. *p < 0.05, **p < 0.01 and ***p < 0.001.

## Results

### USP16 is positively correlated with the c-Myc gene signature

**Fig. 1:**
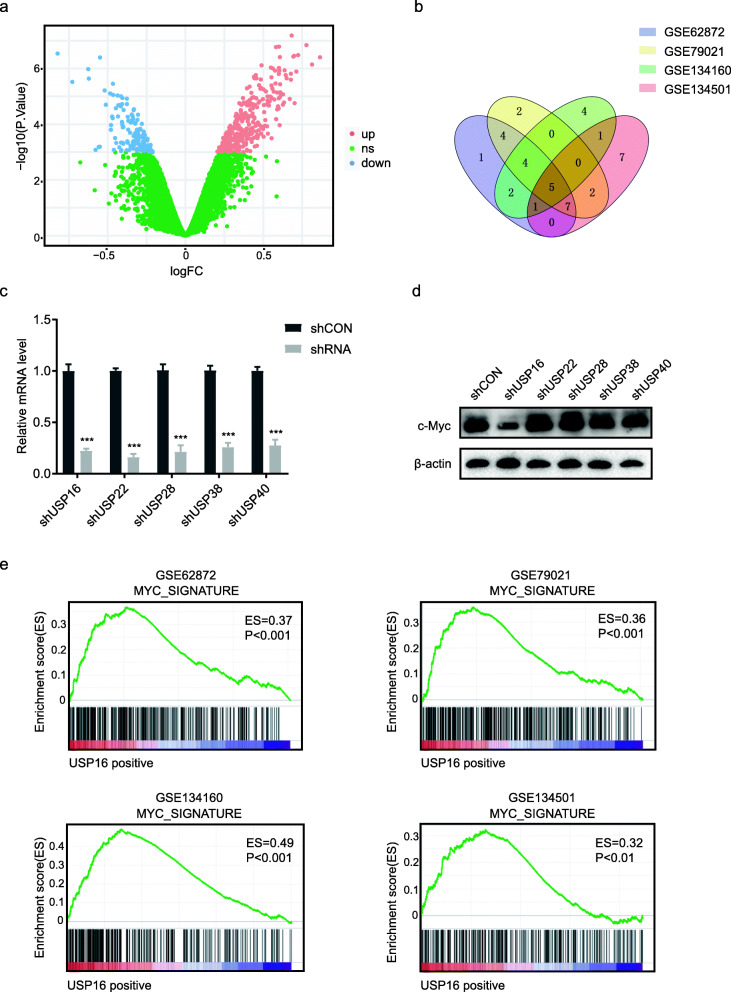
USP16 is highly associated with c-Myc gene signature. **a** A volcano plot presents the differentially expressed genes between control and c-Myc-overexpressing PCa cells. **b** Venn diagram showing USPs that were positively correlated with the c-Myc gene signature in PCa datasets. USP numbers are labelled inside each of the ovals. **c** Knockdown efficiency of shRNAs is shown. **d** Western blot analysis of PC3 cells transfected with indicated shRNAs. **e** Plots indicating a significant positive correlation between USP16 and the c-Myc gene signature in four independent PCa datasets

To explore potential DUBs that may regulate c-Myc signalling, we performed differential expression analysis of control and c-Myc-overexpressing PCa cells (GSE51384), revealing 310 c-Myc mediated up-regulated genes (Fig. 1a and Additional file 2: Table S2). This set of genes was defined as a c-Myc gene signature and then imported into GSEA software for gene set enrichment analysis. Using this dataset, we screened for ubiquitin-specific proteases (USP) that were positively correlated with the c-Myc signature in four publicly available human PCa datasets (GSE62872, GSE79021, GSE134501, and GSE134160). Merging of these four independent analyses revealed a set of five USPs (USP16, USP22, USP28, USP38, and USP40) that consistently were identified across all dataset analyses (Fig. 1b).

Next, we transfected shRNAs of the five USPs into PC3 cells, and the knockdown efficiency of shRNAs was measured by qRT-PCR (Fig. 1c). Analysis of c-Myc protein levels by Western blot revealed that knockdown of USP16 significantly decreased the abundance of c-Myc (Fig. 1d). Taken together, these data suggest that USP16 is strongly associated with the c-Myc signalling pathway and may play an important role in PCa. The positive correlation between USP16 and the c-Myc gene signature in PCa datasets was shown in Fig. 1e.

### Targeting USP16 inhibits CRPC cells proliferation in vitro

**Fig. 2:**
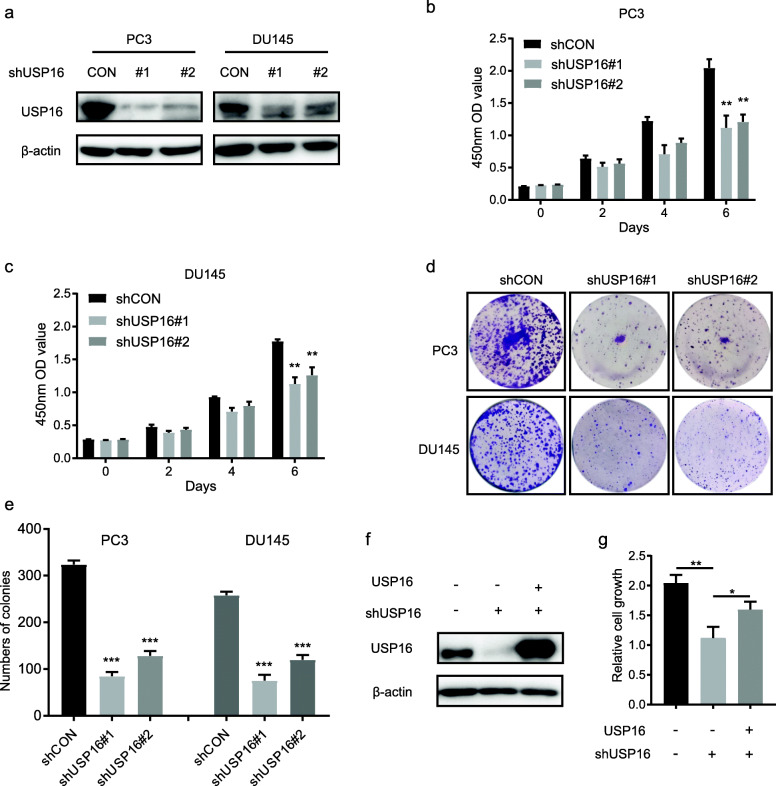
Disrupting USP16 impairs PCa cell viability. **a** The knockdown efficiency of shRNA was confirmed by Western blot; β-actin was used as a loading control. **b** and **c** The viability of PC3 and DU145 cells was measured by CCK-8 assay at the indicated times. **d** and **e** Colony formation assays were performed in PC3 and DU145 cells transfected with the indicated shRNAs and the colony numbers of each group are shown. **f** PC3 cells were infected with control or USP16 (shUSP16#1-resistant) lentivirus as indicated with or without USP16 knockdown. USP16 protein level was measured by Western blot. **g** Relative cell growth was determined using a CCK-8 assay at day 6. Each value represents the mean ± standard deviation of three independent experiments

To better understand the role of USP16 in PCa cells in vitro, we silenced USP16 in two CRPC cell lines: PC3 and DU145 cells, which characterized by androgen-independent growth. The knockdown efficiency of USP16 was confirmed by Western blot (Fig. 2a). Cells proliferation were then analysed using a CCK-8 assay, and the results revealed that knockdown of USP16 markedly reduced cell growth in PCa cells (Fig. 2b and c). Colony formation assays yielded the similar results that USP16 deletion results in significant reductions in cell colony numbers relative to controls (Fig. 2d and e). Concordantly, we found that ectopic expression of USP16 mostly restored the proliferation rate of USP16 knockdown cells (Fig. 2f and g). Collectively, these results indicate that USP16 is necessary for the growth of CRPC cells.

### USP16 knockdown suppresses growth of PCa tumour xenografts

**Fig. 3:**
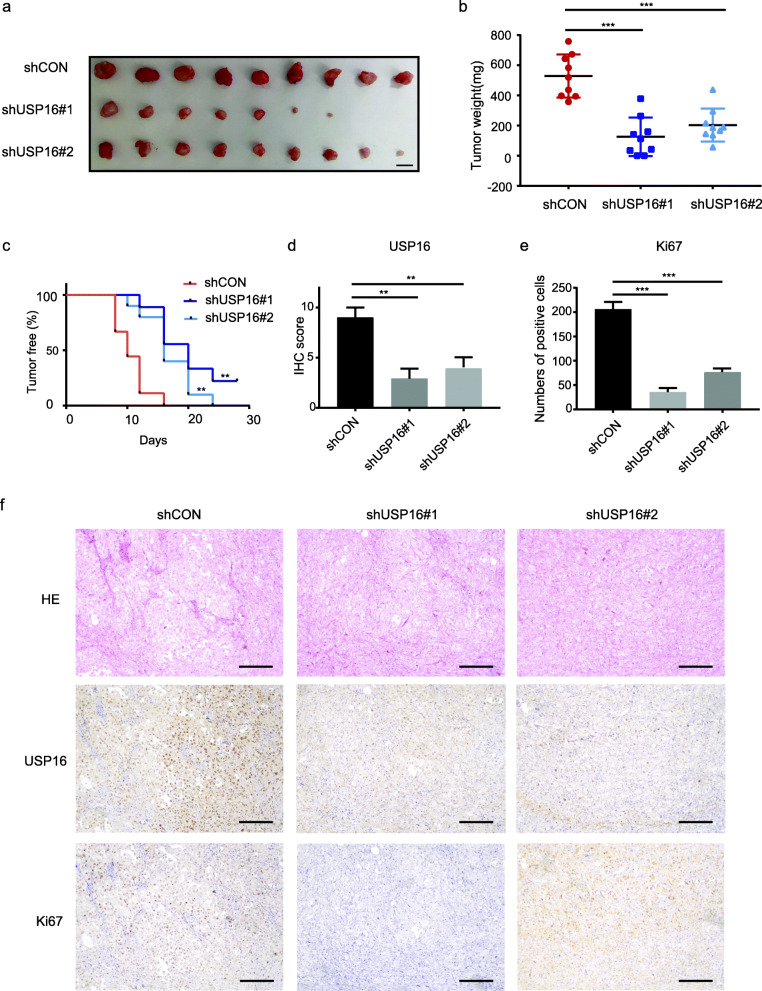
Knockdown of USP16 impeded PCa tumour growth in vivo. **a** PC3-shCON and PC3-shUSP16 cells (5 × 10^5^) were suspended in Matrigel (volume, 1:1) and subcutaneously implanted into nude mice (*n* = 9). Mice were sacrificed 8 weeks later, and the volumes of the xenograft tumours were determined (scale bar: 10 mm). **b** The weights of the xenograft tumours are shown. Error bars represent mean ± standard deviation (Mann–Whitney test; *n* = 9). **c** Kaplan–Meier analysis of tumour onset (log rank). **d** USP16 expression is shown as an IHC score (Mann–Whitney test). **e** Ki67 expression is expressed as the number of positive cells. **f** Haematoxylin-eosin (HE) staining and USP16 and Ki67 IHC staining in tumour xenografts (scale bar: 100 μm)

To further elucidate the role of USP16 in the growth of PCa cells in vivo, PC3 cells stably expressing shRNA targeting USP16 (shUSP16) or vector control (shCON) were subcutaneously injected into 6-week-old male nude mice. After 8 weeks, the mice were sacrificed and the xenografts were extracted for further investigation. The control group xenografts (shCON) were larger and weighed significantly heavier than those in the USP16 knockdown group (shUSP16) (Fig. 3a and b). In addition, the inhibition of USP16 led to a delayed tumour onset in nude mice (Fig. 3c). IHC staining analysis of the xenograft tissues revealed that inhibiting USP16 reduced Ki67 expression, indicating USP16 knockdown impaired the proliferation of PCa cells (Fig. 3d–f). These results demonstrate that inhibiting USP16 significantly suppressed PCa cell growth in vivo.

### USP16 stabilizes c-Myc in a deubiquitination activity-dependent manner

**Fig. 4:**
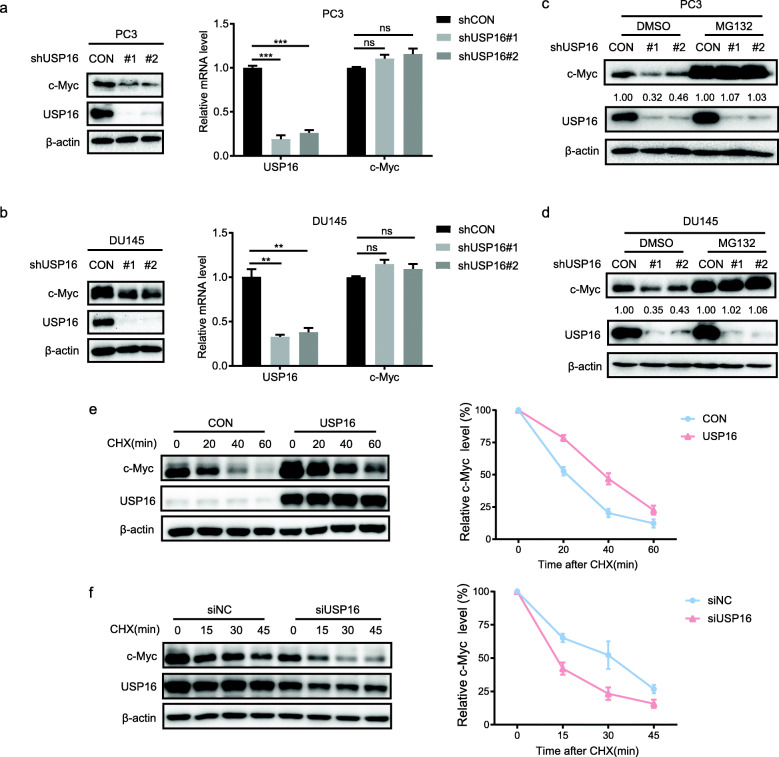
USP16 regulates c-Myc stability at the post-transcriptional level. **a** and **b** Expression of USP16 and c-Myc proteins were measured by Western blot, and mRNA levels of USP16 and c-Myc were detected by qRT-PCR. **c** and **d** PC3 and DU145 cells were treated with 10 μmol/L of MG132 for 6 h and then lysed. **e** and **f** PC3 cells were transfected, treated with 50 μg/ml of CHX, and harvested at the indicated times. The plot illustrates c-Myc band intensities quantified by densitometry

In previous assays, we observed the malignant effects of USP16 in PCa cells, and then we would like to further uncover the underlying mechanisms of how USP16 exert such effects. We found that knockdown of USP16 dramatically reduced c-Myc protein abundance but did not affect its mRNA levels (Fig. 4a and b), suggesting that the regulation of c-Myc by USP16 occurs at the post-transcriptional level. Moreover, treatment with proteasome inhibitor MG132 significantly attenuated the effect of USP16 knockdown on c-Myc protein level (Fig. 4c and d). Next, we examined whether USP16 could regulate the stability of c-Myc using a cycloheximide (CHX) chase assay. PC3 cells were treated with 50 μg/L CHX and c-Myc protein levels were measured at a series of indicated time points. We found that ectopic expression of USP16 enhanced the stability of c-Myc protein, while USP16 knockdown reduced the half-life of c-Myc protein (Fig. 4e and f). These data indicate that USP16 specifically sustains c-Myc stability through the ubiquitination-proteasome pathway.

### USP16 deubiquitinates c-Myc

**Fig. 5:**
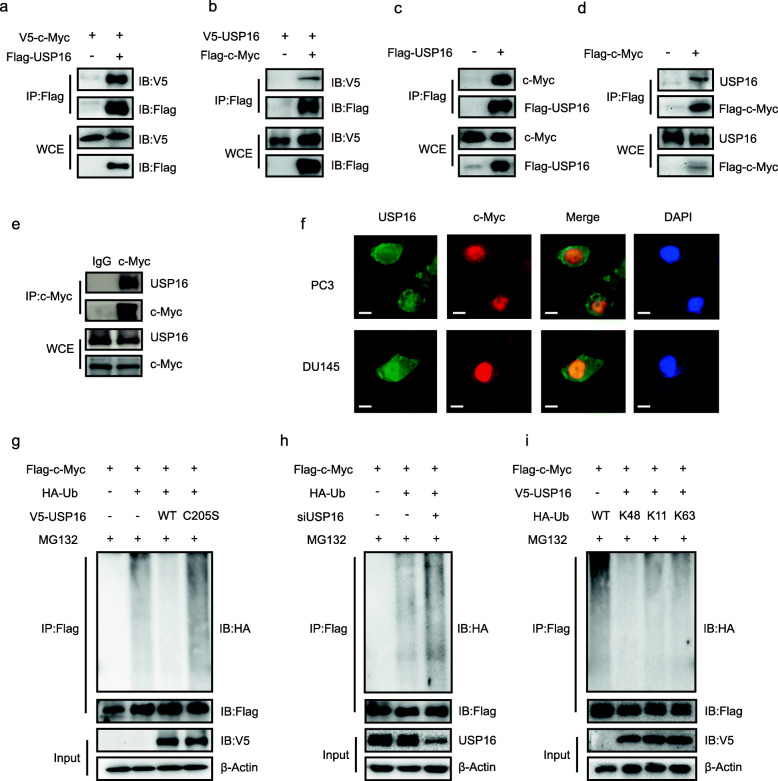
USP16 co-localizes and interacts with c-Myc. **a** and **b** Indicated plasmids were transfected into HEK293T cells alone or in combination, and an anti-Flag antibody was used for immunoprecipitation. IP, immunoprecipitation; IB immunoblotting; WCE, whole-cell extract. **c** and **d** Exogenous USP16 or c-Myc was immunoprecipitated from PC3 cells using an anti-Flag antibody. Endogenous c-Myc or USP16 was analysed by Western blot. **e** Interaction between endogenous c-Myc and USP16 in PC3 cells was analysed by Western blot. **f** Immunofluorescent staining of USP16 and c-Myc in PC3 and DU145 cells. Scale bar, 10 μm. **g** Flag-c-Myc and HA-ubiquitin were co-transfected with v5-USP16 or v5-C205s, and the ubiquitination of c-Myc was measured using an in vivo ubiquitination assay. Precipitates and WCEs were analysed by Western blot. **h** Control siRNA (siNC) or siUSP16 was transfected into HEK293T cells. After 48 h, cells were transduced with Flag-c-Myc and HA-Ub for 24 h and then harvested. **i** HEK293T cells were transfected with v5-USP16, Flag-c-Myc, and different forms of HA-ubiquitins. Cell lysates were immunoprecipitated with an anti-Flag antibody and ubiquitination levels were measured using an anti-HA antibody

To explore the functional links between USP16 and c-Myc, we transfected Flag-tagged and V5-tagged plasmids into HEK293T cells, followed by immunoprecipitation with an anti-Flag antibody. Ectopically expressed USP16 was found to significantly interact with c-Myc and vice versa (Fig. 5a and b). Furthermore, USP16 protein was detected when Flag-c-Myc was immunoprecipitated by Flag antibody, and inversely c-Myc was detected when Flag-USP16 was immunoprecipitated in PC3 cells (Fig. 5c and d). The interaction between endogenous c-Myc and USP16 was also demonstrated in PC3 cells (Fig. 5e). Next, we confirmed the co-localization of USP16 and c-Myc in PC3 and DU145 cells using immunofluorescent staining (Fig. 5f). Together, these data indicate that USP16 both interacts and co-localizes with c-Myc.

To identify whether USP16 serves as a DUB of c-Myc, HEK293T cells were transfected with plasmids encoding HA-ubiquitin and Flag-c-Myc with wild-type USP16 or its catalytically inactive mutant USP16-C205S and treated with MG132. As shown in Fig. 5g, the wild-type USP16, but not USP16-C205S, markedly reduced the ubiquitination of c-Myc. Besides, the knockdown of USP16 significantly enhanced the polyubiquitination of c-Myc (Fig. 5h).

Next, we identified which polyubiquitin modification of c-Myc protein was regulated by USP16. HEK293T cells were transfected with v5-USP16 and Flag-c-Myc, along with one each of the different HA-ubiquitins (WT, K11, K48, or K63). Cell lysates were then immunoprecipitated with an anti-Flag antibody and subjected to immunoblotting analysis using an anti-HA antibody. The results revealed that the K48-linked ubiquitination of c-Myc was substantially reduced by USP16 (Fig. 5i).

### USP16 regulates PCa cell growth through c-Myc

**Fig. 6:**
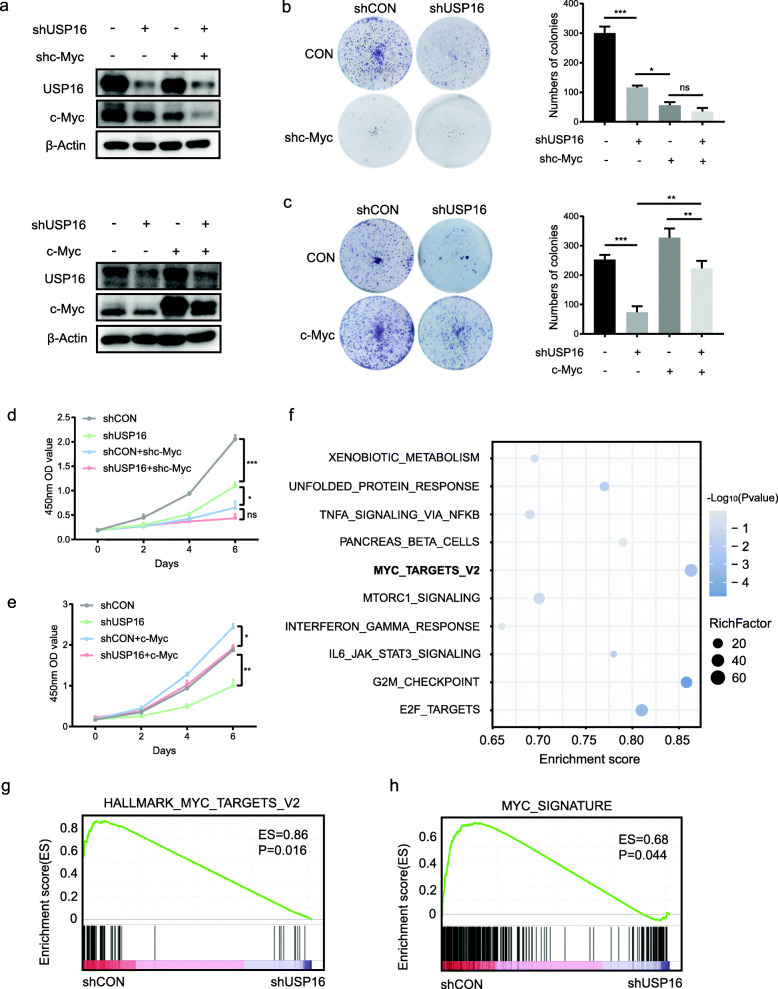
USP16 regulates PCa cell proliferation via c-Myc. **a** Expression of USP16 and c-Myc was evaluated by Western blot. **b** and **c** PC3 cells were transfected with the indicated plasmids and cell growth was measured by colony formation assay. **d** and **e** Cell viability was determined by CCK-8 assay. **f** Bubble plot of GSEA results. **g** and **h** Gene expression profiles of PC3 cells with or without knockdown of USP16 based on the Myc target and signature gene set

c-Myc is an oncoprotein involved in cell proliferation, and overexpression of c-Myc is known to enhance the viability of several cancer cells [24]. Given that USP16 regulates the stability of c-Myc, we examined whether USP16 regulates cell growth through c-Myc. We either disrupted or overexpressed c-Myc under conditions of USP16 knockdown. The protein levels of USP16 and c-Myc were measured using Western blot (Fig. 6a). Colony formation assays results suggest that c-Myc knockdown could abolish the effect of USP16 knockdown in terms of both cell proliferation and growth (Fig. 6b). Moreover, c-Myc overexpression restored the proliferation and colony formation abilities of USP16 silenced cells (Fig. 6c). These findings were further confirmed by CCK-8 assays (Fig. 6d and e).

We then performed RNA-Seq analysis of PC3 cells with or without USP16 knockdown and the gene expression profiles were analysed by GSEA (Fig. 6f). Consistent with the previous screening results of published datasets, the Myc targets and Myc gene signature gene set were significantly enriched in the control group (Fig. 6g and h). Together, these data indicate that USP16 regulates PCa cell proliferation mainly throughs stabilizing c-Myc.

### USP16 expression is elevated in prostate cancer

**Fig. 7:**
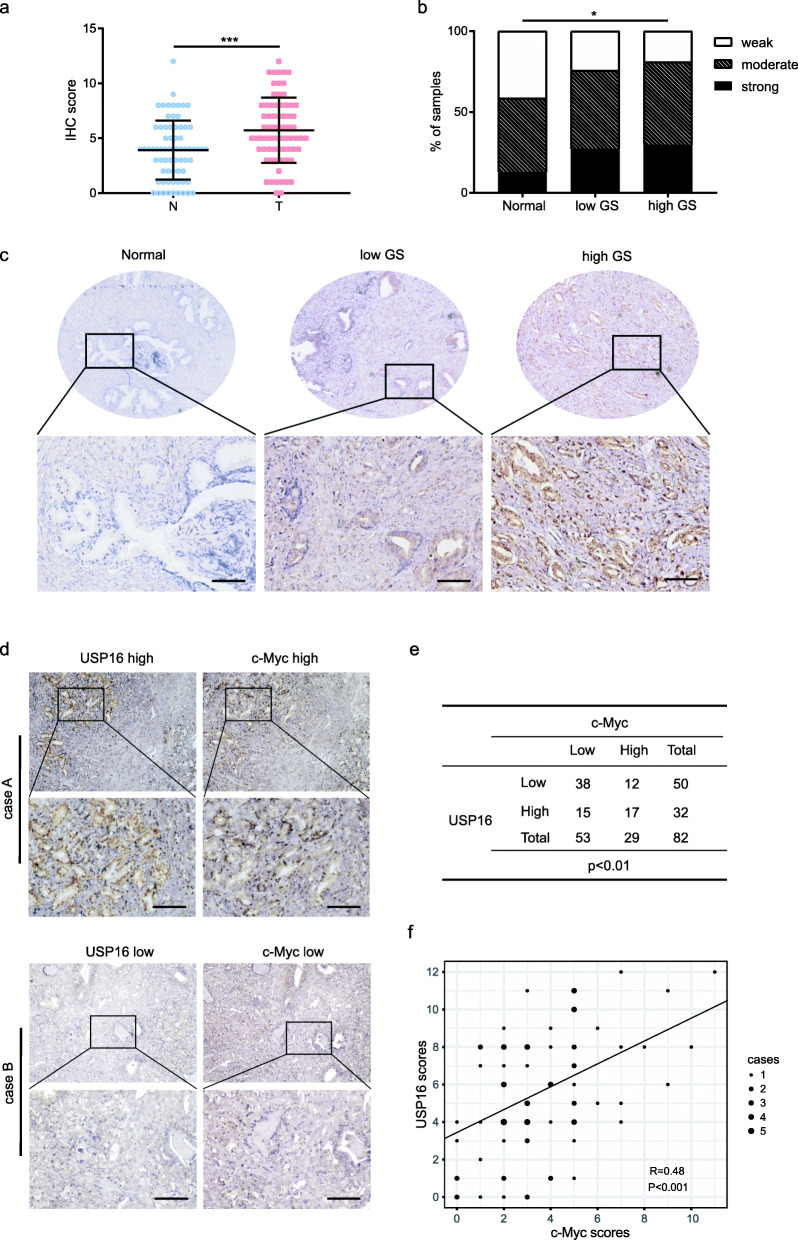
USP16 expression is upregulated in prostate cancer. **a** Expression of POH1 in normal prostate tissues (N) and PCa tissues (T) were examined by IHC (Mann-Whitney test, *p* < 0.001). **b** Distribution of USP16 expression among prostate samples as indicated (N, normal prostate; low GS, Gleason scores ≤3 + 4; high GS, Gleason scores ≥4 + 3; weak, IHC scores ≤4; mod, 4 < IHC scores ≤8; strong, IHC scores > 8) (χ2 test; *p* < 0.05). **c** Representative images of prostate samples (scale bar: 100 μm). **d** Case A: a representative sample with high USP16 and c-Myc staining. Case B: a representative sample with low USP16 and c-Myc staining. Scale bar: 100 μm. **e** Quantification of the staining revealed a statistically significant correlation. (χ2 test; *p* < 0.01); **f** Correlation between USP16 and c-Myc expression in PCa samples are shown (Spearman correlation test; *p* < 0.001). R indicates the correlation coefficient

Next, we sought to confirm our results in clinical samples. We characterized USP16 expression in PCa (*n* = 70) and adjacent normal tissues (*n* = 70) via IHC staining. The staining scores of normal prostate tissues were markedly weaker than PCa tissues (Fig. 7a). Furthermore, we noticed that the USP16 staining scores were significantly correlated with the Gleason scores (χ2 test; *p* < 0.05; Fig. 7b and c), which indicates the essential role played by USP16 in PCa development.

To further assess the association between USP16 and c-Myc in PCa, we detected the expression of USP16 and c-Myc using tissue microarrays containing 82 human PCa tissues. Consequently, a positive correlation was found between the staining scores of USP16 and c-Myc (Fig. 7d-f). Thus, these results revealed the clinical relevance of USP16-mediated regulation of c-Myc in PCa development.

## Discussion

Deubiquitination is a widespread post-translational modification of proteins that affects diverse biological processes. DUBs can remove ubiquitin from substrates and thus stabilize target proteins, as well as regulate their subcellular localization and function [25]. Ubiquitin-specific proteases (USPs) account for the majority of DUBs and are involved in a variety of cellular processes including cell proliferation, differentiation, and metabolism [26]. Furthermore, USPs are significantly correlated with the occurrence of numerous cancers including PCa. For example, USP10 interacts with G3BP2 to block p53 signal transduction, leading to poor prognosis in prostate cancer [27]; USP44 promotes the development of prostate cancer by stabilizing EZH2 [28], and USP2a enhances c-Myc expression via microRNA-related regulation and thus promotes tumourigenesis [29]. Moreover, the AR protein, which is frequently dysregulated in PCa, has been reported as a substrate for several USPs including USP12 [30], USP22 [31], USP26 [32], and USP7 [33]. Besides controlling AR and ARv7, USP7 is also in charge of the stability of the CCDC6 gene product, a tumor suppressor whose impairment induces a BRCAness-like phenotype which associates to PARP inhibitors sensitivity in prostate and urothelial cancer [34–36].

In PCa, c-Myc plays a key role in disease progression [16], and c-Myc expression is positively correlated with advanced histologic grade and poor prognosis. Hubbard et al. found that the combination of Myc activation and Pten loss could result in lethal prostate cancer [37]. However, despite this strong evidence linking c-Myc and disease pathology, few effective treatments have targeted c-Myc due to its structure and location [38]. As a transcription factor, c-Myc lacks enzymatic activity, making it difficult to target by small molecules. Additionally, c-Myc is predominantly located in the nucleus, thereby rendering monoclonal antibody-based therapies impractical [39]. To overcome this limitation, researchers have instead focused on targets upstream of c-Myc, such as BET bromodomain inhibitors [40], which have demonstrated preclinical efficacy in models of c-Myc-driven CRPC and shown considerable promise as a therapy for PCa [41].

Given the strong transcription-promoting activity of c-Myc, it is not surprising that c-Myc abundance is tightly controlled by multiple mechanisms [42]. One of the most significant mechanisms maintaining appropriate c-Myc protein levels is the ubiquitin–proteasome system (UPS). In the present study, we found that the targeted disruption of USP16, but not other deubiquitinases of c-Myc, reduced c-Myc protein levels in PCa, indicating that DUBs have different functions in different cancers.

It has been reported that USP16, one of the deubiquitinases of Histone H2A, is required for removing ubH2A-mediated repression during the differentiation of embryonic stem cells [23]. Besides, USP16 can regulate hematopoiesis and the function of hematopoietic stem cells [43] . However, the role of USP16 in tumourigenesis remains poorly understood. Here, we demonstrated that USP16 knockdown notably impaired PCa cell proliferation both in vitro and in vivo. In addition, we found USP16 regulates PCa cell growth through stabilizing c-Myc. Furthermore, USP16 expression was upregulated in PCa tissues and correlated with PCa pathological grade and c-Myc expression, suggesting that USP16 plays a critical role in PCa tumourigenesis and could be a therapeutic target of PCa.

As indicated in Fig. 6f, USP16 knockdown may result in potential effects independent from c-Myc, such as G2M checkpoint and E2F targets. It has been reported that the silence of USP16 in Hela cells could decrease the proportion of cells at G2/M partially due to the defects of H2A deubiquitination during G2/M phase progression [44]. In addition, HSCARG, a negative regulator of H2A ubiquitination, is necessary for cell proliferation and the deletion of HSCARG could activate checkpoint signaling and impair cell viability [45]. Besides, USP21 could promote transcription by deubiquitylating ubH2A in vitro [46]. Therefore, the deubiquitination of H2A by USP16 may also play a role in prostate cancer cell proliferation to some extent, which remains further investigated. On the other hand, c-Myc target genes include critical positive cell cycle regulators like cyclin-dependent kinases, cyclins and E2F transcription factors [47]. Moreover, c-Myc promoter contains an E2F binding site which is crucial for c-Myc expression [48]. In brief, c-Myc has synergistic effect with E2F and the specific mechanism between c-Myc and E2F awaits further study. Additional work will be required to determine which part of c-Myc transcriptional network could be regulated by USP16 so that we could find the key genes or pathways involving transformation of CRPC or NEPC. Besides, it remains to explore whether c-Myc signalling could activate USP16 expression, resulting in a positive feedback loop that further promotes tumourigenesis.

## Conclusion

Our study identified USP16 as a novel deubiquitinase of c-Myc and revealed its important role in the development and progress of PCa. RNA-seq data from PCa cells demonstrated that USP16 was strongly associated with c-Myc expression signatures and downstream targets. And the positive correlation between USP16 and c-Myc was further confirmed by IHC staining of tissue microarray. Given the essential role played by c-Myc in PCa, targeting USP16 may represent a potential therapy for PCa treatment.

## Supplementary Information

**Additional file 1: Table S1.** qPCR primers and shRNAs sequences.

**Additional file 2: Table S2.** c-Myc gene signature.

**Additional file 3: Fig. S1.** Films of long and short exposure in cycloheximide chase assay.

## Data Availability

The datasets supporting the findings of this study are indicated in the article.

## Abbreviations

AR: Androgen receptor
CCK8: Cell counting kit-8
CHX: Cycloheximide
CRPC: Castration-resistant prostate cancer
DUB: Deubiquitinase
GS: Gleason scores
GSEA: Gene set enrichment analysis
IHC: Immunohistochemistry
IP: Immunoprecipitation
PCa: Prostate cancer
qRT-PCR: Quantitative real-time polymerase chain reaction
UPS: Ubiquitin proteasome system
USP16: Ubiquitin-specific protease 16
USPs: Ubiquitin-specific proteases
WCE: Whole-cell extract
