# Supplementary material for: USP16 regulates castration-resistant prostate cancer cell proliferation by deubiquitinating and stablizing c-Myc
Source: J Exp Clin Cancer Res. 2021 Feb 5;40:59. doi: 10.1186/s13046-021-01843-8 (PMC7866668; doi:10.1186/s13046-021-01843-8)
Supplement: Supplementary file 1 — Additional file 1: Table S1. qPCR primers and shRNAs sequences. [file 13046_2021_1843_MOESM1_ESM.docx]

**Table S1.** qPCR primers and shRNAs sequences

| Primer | Sequence |
| --- | --- |
| USP16 Forward | 5′-TCTGTCGCCGTGGATTGTTA-3′ |
| USP16 Reverse | 5′-TGTGTCTGCACACAGGTTCT-3 |
| USP22 Forward | 5'-AGGCCATGGACGCCGA-3' |
| USP22 Reverse | 5'-ACTTGGCCTTGCGCTTGC-3' |
| USP28 Forward | 5'-CACAAGCTCCTGGATTGGCT-3' |
| USP28 Reverse | 5'-AGAGGATACTGGCCGAAGGT-3' |
| USP38 Forward | 5'-ACGAGCAGGTTTCCAAAGGA-3' |
| USP38 Reverse | 5'-CTGGTGGGTCGTTGTCATCA-3' |
| USP40 Forward | 5'-GGAACTGGCTTCTCGTTCCA-3' |
| USP40 Reverse | 5'-AACTCTGCCCCAAGACGAAG-3' |
| c-Myc Forward | 5'-AAAGGCCCCCAAGGTAGTTA-3' |
| c-Myc Reverse | 5'-TTTCCGCAACAAGTCCTCTT-3' |
| GAPDH Forward | 5'-AGCCACATCGCTCAGACAC-3' |
| GAPDH Reverse | 5'-CCCTGTCTGTCCTCTGTAGC-3' |
| shUSP16#1 sense | 5′-GCCAGAAGAAATCATGTTTAT-3′ |
| shUSP16#1 antisense | 5′-ATAAACATGATTTCTTCTGGC-3 |
| shUSP16#2 sense | 5'-CGGTGATATTCCACAAGATTT-3' |
| shUSP16#2 antisense | 5'-AAATCTTGTGGAATATCACCG-3' |
| shUSP22 sense | 5'-CACAAGCTCCTGGATTGGCT-3' |
| shUSP22 antisense | 5'-AGAGGATACTGGCCGAAGGT-3' |
| shUSP28 sense | 5'-GCTCTAATGATGGGATCAAAT-3' |
| shUSP28 antisense | 5'-ATTTGATCCCATCATTAGAGC-3' |
| shUSP38 sense | 5'-CGTCTAATACTATGACTGTTA-3' |
| shUSP38 antisense | 5'-TAACAGTCATAGTATTAGACG-3' |
| shUSP40 sense | 5'-CGGAGATACTATTGGTGTTAA-3' |
| shUSP40 antisense | 5'-TTAACACCAATAGTATCTCCG-3' |
