# Supplementary figures and images for: USP16 regulates castration-resistant prostate cancer cell proliferation by deubiquitinating and stablizing c-Myc
Source: J Exp Clin Cancer Res. 2021 Feb 5;40:59. doi: 10.1186/s13046-021-01843-8 (PMC7866668; doi:10.1186/s13046-021-01843-8)

**a**

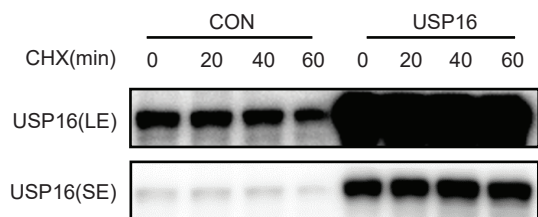

**b**

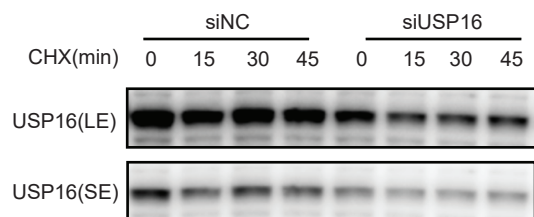

Supplement: Supplementary file 3 — Additional file 3: Fig. S1. Films of long and short exposure in cycloheximide chase assay. [file 13046_2021_1843_MOESM3_ESM.pdf]
